# Don't ask don't tell: Battered women living in Sweden encounter with healthcare personnel and their experience of the care given

**DOI:** 10.3402/qhw.v9.23166

**Published:** 2014-02-26

**Authors:** Darcia Pratt-Eriksson, Ingegerd Bergbom, Elisabeth D. Lyckhage

**Affiliations:** 1 Institute of Health and Care Sciences, Sahlgrenska Academy, University of Gothenburg, Göteborg, Sweden; 2 Karolinska University Hospital, Huddinge, Sweden; 3 Institute of Nursing, Health and Culture, University West, Trollhättan, Sweden

**Keywords:** nursing, intimate partner violence, emergency, uncaring, suffer

## Abstract

In recent years there has been increased intimate partner violence (IPV) toward women. Research on the care provided to victims of IPV is limited. The purpose of the study was to gain a deeper understanding of women's lived experience of IPV and their encounters with healthcare professionals, social workers, and the police following IPV. A phenomenological hermeneutic method inspired by the philosophy of Paul Ricoeur was used. The method is based on text interpretation and gives voice to women's lived experience. Twelve women living at a women's shelter in Sweden narrated their IPV experiences. The study revealed that the women experienced retraumatization, uncaring behaviors, and unendurable suffering during their encounter with healthcare professionals. They were disappointed, dismayed, and saddened by the lack of support, care, and empathy. Nurses and other healthcare professionals must understand and detect signs of IPV as well as provide adequate care, as these women are vulnerable. IPV victims need to feel that they can trust healthcare professionals. Lack of trust can lead to less women reporting IPV and seeking help.

Gender equality is one of the cornerstones of Swedish society. Despite Sweden being acknowledged as one of the world leaders in equality, intimate partner violence (IPV) is a growing problem that needs to be addressed (The Swedish Institute, 2011). The most common perpetrators of violence against women are husbands and male partners (Dahlberg & Krug, 2002). According to Häggblom and Möller (2007), IPV is still a hidden public health issue and may be underestimated by family members, politicians, health workers, social service, the police, the media, and other institutions. According to The Swedish National Council for Crime Prevention (2009), there has been a 34% increase in reports of violence against women over the past 10 years. The Swedish National Board of Health and Welfare stated that violence against women does not only entail suffering and ill-health for those directly involved but also leads to major financial burden on society due to the cost associated with medical care, the judicial system, the social services, social insurance, unemployment, and production loss (Envall, Eriksson, & Marnell, 2006). The National Centers for Disease Control and Prevention (2003) estimates that the cost of intimate partner rape, physical assault, and stalking totaled $5.8 billion each year for direct medical and mental healthcare services and lost productivity from paid work and household chores. IPV is the cause of 30% of female homicides in the United States and has multiple negative consequences. Although these facts are known, few studies have focused on the ability of the healthcare sector to prevent IPV and take care of victims.

There are many studies on violence against women that have established the prevalence of IPV as well as the cost and type of injuries (Campbell, Sharps, Gary, Campbell, & Lopez, 2002; Campbell & Soeken, 1999; Haddad, Shotar, Younger, Alzyoud, & Bouhaidar, 2011). According to earlier findings, women are mainly exposed to a combination of violent acts and coercive control tactics such as threats and intimidation while men are the prime perpetrators of such violence in heterosexual relationships. Traditional gender norms, power structures, and gender inequalities between men and women are core factors that give rise to coercive control tactics (Stark, 2010). IVP is connected to the society's prevailing gender order including power imbalances associated with traditional gender roles, acculturation, socioeconomic status, and education level (Watson, 2010). Dependency, submission, and transgression add to dominance, which in turn contributes to IPV (Choi & Ting, 2008). Using a power inequality perspective, an imbalance of economic resources within the couple that underlies the use of violence can be illuminated. An early study showed that IVP is less likely to occur in societies where women have strong and close bonds to their families (Kruttschnitt, 1995).

However, few investigated the women's experiences of the care they received following IPV. Several studies have demonstrated that IPV has negative long-term health consequences for the victims, even after it has ended (Campbell & Soeken, 1999; Campbell et al., 2002; Haddad et al., 2011). IPV results in physical injury, psychological trauma, and sometimes death (Kernic, Wolf, & Holt, 2000). The role of caregivers should involve posing questions, confirming, documenting, and referring to specialist care. Appropriate questions about violence should be developed. There is also a lack of knowledge among nurses and other healthcare professionals about women's lived experience of IPV and the care provided following the violence (Stenson, 2004). Häggblom and Möller (2007) found that professionals who were supportive helped the women while other professionals revictimized the women. The results showed that some of their informants had felt insulted by health and social workers who “offered haphazard services” (p. 169). The women described nurses who had understood them, while others were nonresponsive, making them feel ashamed. Also, when meeting social workers, their experiences ranged from meeting helpful and compassionate social workers to those with an unsympathetic attitude. However, regarding encounters with the police, the women were pleasantly surprised by their attitudes. Healthcare professionals must acknowledge and address IVP as a growing problem. There is a substantial body of knowledge about the consequences of IPV, which will be discussed in the present study in the light of women's long-term care needs. The purpose of this study was to gain a deeper understanding of women's lived experience of IPV and their encounters with healthcare professionals, social workers, and the police following IPV.

## Methods

This is a descriptive and exploratory study with a qualitative approach. Lindseth and Norberg's phenomenological hermeneutic method (2004), which was inspired by the philosophy of Paul Ricouer (1976), was used. Ricouer (2005) demonstrated that the narrative is one way of giving voice to lived experience. Interpretation is a particular form of understanding applied to written lived experience. When reading the text, the interpreter moves dialectically between various attitudes, that is, guessing, distanciation, explanation, validation, understanding, and finally mediation of the findings. The text must be read carefully in order to grasp its full meaning and understand what it mediates (Ricouer, 1976). The naïve reading is seen as an initial conjecture that should be validated or invalidated by the subsequent structural analysis, which is guided by the naïve reading and involves identifying meaning units (segmentation) and integrating the parts at various levels into a whole.

### Setting and participants

The sample consisted of 12 women living at a women's shelter in Stockholm, Sweden, who were asked to narrate their experiences of IPV and encounters with healthcare professionals, social workers, and the police.

Inclusion criteria were: (1) women aged 18 years and above, (2) good knowledge of the English or Swedish language, and (3) women who are/were victims of violence from a cohabitating/former partner or husband. The women's ages ranged between 23 and 56 years, with a median age of 28 years. Only one woman had university education, two women had a 2-year college diploma, and the other nine had high school diplomas. Three women were born in Sweden, three were naturalized, and six were immigrants. In terms of IPV, two women who were married were abused by their husbands, one was still being abused by a cohabitating partner, and nine were abused by their former partner.

### Data collection

Data were collected by means of interviews. The first author asked the women to narrate their thoughts, feelings, and experiences in their own words. Follow-up questions such as: ‘What do you mean?’ and ‘Can you tell me more?’ were posed to deepen the responses and increase the richness of the data. The interviews lasted for approximately 45–60 min.

### Data analysis

All authors participated in the analysis process, which took the form of a back and forth movement. It comprised the following steps: naïve interpretation, structural analysis, and comprehensive understanding. The narratives were analyzed in three phases of interpretation:

1. The first step consisted of reading the 12 narratives using a phenomenological approach that allows the text to speak, including guesses that motivate further examination of the text. The reading enables an intuitive understanding of the whole as a starting point for the following steps in the interpretation.
2. The second step was a thematic structural analysis guided by the naïve reading. Each sentence was analyzed and meaning units were identified. Similar units were grouped into subthemes and themes after which they were condensed and reflected on in terms of similarities and differences. These units were carefully read through again and reflected on with reference to the naïve understanding. The condensed themes, that is, the essential meaning of each unit, were expressed as concisely as possible in everyday language (Ricouer, 1976).
3. Finally, a comprehensive understanding was developed taking the authors’ preunderstandings, the naive reading, the structural analysis, and the literature into account. The authors’ preunderstandings comprise knowledge and experience of working in shelters for abused women and children, gender and trauma research, and as nurses in acute care settings.

### Ethical considerations

The authors were aware that victims of IPV are a vulnerable group. Glass, Wellings, Branigan, and Mitchell (2000) stated that research could be deemed sensitive if it requires disclosure of personal behaviors or attitudes that would normally be kept private as they might cause offense, lead to social censure or disapproval, and/or which the respondent may find distressing to express. To ensure that each woman was aware of the sensitivity of disclosing details of the violence, staff from the shelter informed them about the nature of the study and that it could trigger negative emotions. The potential respondents received both oral and written information about the purpose of the study, the interview process, confidentiality, anonymity, and their right to decline participation or withdraw at any time. They were informed that some quotations would be included when reporting the study in a scientific journal. Consent was granted orally. The interviews were audiotaped with the women's permission. Before the study was conducted, the Ethics Research Committee at Gothenburg University, Sweden, granted approval (No: 009-06), and the principles of the World Medical Association's Declaration of Helsinki were adhered to (World Medical Association, 2013).

## Results

### Naïve understanding

Being a battered woman means being a woman who finds it hard to be noticed and taken seriously. To encounter healthcare professionals, social workers, and the police is difficult, as she is being humiliated and hurt by the professionals’ treatments and attitudes. Her hope that the one who hurt her shall receive a penalty is not fulfilled. Instead of being comforted, she is being blamed, and instead of being listened to she is made to feel that she is needlessly occupying their time. It also means that she is to return home without having received the help she craved for and regret that she said that she was beaten.

### Structural analysis

The women's reflections were grouped into three main and six subthemes: Unsupportive communication arose when the women perceived being treated as unworthy without opinions or value.

#### A feeling of being betrayed by the system

The women felt their confidence waning. They had believed that reporting the assault would end their suffering and create a better situation.

*No one cared about my suffering*. The narratives revealed the women's experience of feeling betrayed by the system and their sense of being in conflict with the authorities. They realized that there were many boundaries between healthcare providers, the social services and the police. One woman recalled: *I feel the system has failed me*. Many had to justify the fact that they were too ill to go to work or take care of their children and felt they had to struggle to make the Swedish Social Insurance Agency, the social services and their physician listen to and believe them. They had the impression that healthcare professionals devoted more attention to diseases, ailments, and accidents, than to victims of IPV. One woman explained:

Often you really have to stamp your foot to get a little help or you have to be lying dead in the emergency room for them to help you! It's important for healthcare workers to screen for violence, it's important to find out how we feel.

*Justifying the violence*. Several women stressed their dismay at the way they were treated. They felt as if they were reliving the violence and in some cases started to believe that the treatment to which they were subjected by their abuser was justified. Another woman stated:

I feel like I'm caught up in a catch-22. The Swedish Social Insurance Agency questioned how ill I was. First the physician had to certify that I'm very ill for me to get a sick leave, and then they question how ill I really am!

The women's narratives revealed that they were of the impression that the police did not understand them. Everything stood still, time felt everlasting and their long wait to be rescued seemed endless. One woman narrated how she felt: *Economically I haven't had the energy to apply for economic support because it's horrible, just the fact I have to sit and defend myself.*

The women became distressed when they heard that their case had been dropped due to lack of evidence and many of them perceived the investigation process as a new assault.

#### Feelings of not being taken seriously and respected

In this theme, the women felt that they had lost their identity and that there was no place for them in healthcare. They felt degraded and lacked normality in their lives.

*Fear of losing autonomy*. A sense of not being truthful and not taken seriously was described as problematic by the women, who narrated about their need for respect and understanding of their situation from healthcare professionals, the police, and social workers. Several women with a foreign background felt especially ignored: One woman described her visit to the emergency department:

I felt degraded both as a foreigner and as a woman. I think that some of the staff looks down on us as if it's our fault as women that this happened. One male physician asked me if alcohol was involved during the violence. It was a male physician who looked after me and my daughter, and I was obliged to answer his question. I wanted someone to listen to me and give me support. In future I will make sure that I only encounter female healthcare professionals!

*Degraded to nothing*. Some narratives conveyed that the women felt that they were in the way, seen as a nuisance who wasted staff time, and created unnecessary problems. One woman described a visit to the Emergency department:

When the physician came she was so cold. She could have pretended that she understood my situation. When I asked her to put me on sick leave, she asked me why. How could she ask such a question? I thought physicians had experience of situations like this but obviously not.

Another woman disclosed the humiliation she felt as a result of her encounter with the social services:

The social worker thought I should be grateful for the help I had received from social welfare, because I wouldn't have had the same help in my own country. I didn't get any help; I am disappointed.

Women with a minority background and native women felt equally mistreated. They also felt questioned and were treated with suspicion. Another woman narrated how she felt wrongfully treated: *My husband should not be allowed to be present? Today when I re-read my medical records, I am shocked by what it says [there].*

#### Feelings of uncaring attitudes

In this theme, the women reflected upon the care provided and stated that they had a poor relationship with the healthcare professionals. The women felt that the healthcare professionals were uncaring, lacked empathy, patience, and had an unfriendly attitude.

*Losing hope*. Most women were of the opinion that disclosing the violence was unhelpful and pointless. They had the impression of being cutoff from help and work as well as experiencing a lack of financial support. Some women reported feeling stuck and being kept in limbo. Several had thought that reporting the crime would solve all their troubles and that their problems would disappear. Instead, they felt hurt, lonely, and psychologically distressed. Another woman said: *I wish I had received more help from the police, not waiting for six months before they did a search to find my valuables*. One woman revealed:

*First he* got 1,5 years *in prison for gross violation, then he appealed the case to the High Court and they reduced the sentence to six months*. *I just don't understand the system, how is this possible? This made me fall apart.*

Inadequate information about their case and of the barriers in communication procedures lengthens the women's suffering. One woman narrated: *No I didn't receive any help from the social. It takes such a long time before anything happens*. Another woman voiced her experience: *They said that since there isn't any proof the case will be closed*.

*Feeling neglected and invisible*. The women's narratives revealed that they felt ignored, that the care was in a hurried manner, that they had no control over the care provided, and struggled to be seen and heard. They also complained about lack of follow-up, referrals, as well as poor information and awareness. One woman explained:

It's important that the staff ask the woman if she ever experienced abuse. I was raped repeatedly by my husband and bled many times from my vagina. I wasn't allowed to contact the healthcare centre and forced to have a baby born out of rape, so it's important that staff inform women of the help available.

Most women talked about the impact of their encounters with healthcare professionals and how they felt unsafe, powerless, and voiceless. Another woman told of her experience with healthcare: When I slept at the hospital with my daughter, no one came and spoke to me. I wanted someone who could listen and give me support.

### Comprehensive understanding

The final step in the process of analysis according to the phenomenological hermeneutic method strives to comprehend the “parts” to form a deeper understanding of the “whole.” It points to a possible way of being-in-the-world, unfolded in the face of the text. For this study, the comprehensive understanding is: The meaning of being a woman who has experienced violence in an intimate relationship is tantamount to having being twice betrayed. To be neglected means a feeling of being *retraumatized and experiencing an* existential loneliness when her suffering is ignored by health professionals, social workers, and the police. Having exposed herself without any protection and care leaves her all by herself, with no way back and an unclear and precarious future. Her shame and guilt leaves her with nothingness. This way of being can be seen through the lens of the reflections of Eriksson's (2006) theory of suffering as an essential content in every human being's struggle with suffering and battle to survive, that is, to continue the struggle or resign and reject life.

## Discussion

The purpose of the current study was to gain a deeper understanding of women's lived experience of IPV and their encounters with healthcare professionals, social workers, and the police following IPV. IPV is a growing problem all over the world. The findings in our study indicate several flaws in the quality of the care provided to vulnerable women. The women felt wrongfully treated when seeking help from healthcare professionals, social workers, and the police. Our findings are consistent with a growing body of literature indicating that the credibility of such women is questioned and that they perceive an uncaring attitude (Battaglia, Finley, & Liebschultz, 2003; Häggblom & Möller, 2007). The women had feelings of uncaring attitudes, when longing for care and to be treated kindly. Failure on the part of healthcare professionals to treat patients with dignity and respect created even greater suffering. According to Eriksson (2006), both suffering and health are an essential part of every human being. To struggle with suffering and battle to survive, that is, to continue the struggle or to resign and reject life. Causing the other to suffer always implies violation of her/his dignity, failure to confirm her/his value as a human being, and a denial of one's own holiness. The women in our study experienced an existential loneliness when their suffering was ignored by health professionals, social workers, and the police. The experience of not being taken seriously can cause unendurable suffering. The dignity of women must not only be maintained during short meetings at emergency departments but also be restored, which requires caring relationships over a long period. Häggblom and Möller(2007) and Payne (2007) stated that healthcare staff could inadvertently foster revictimization. Our findings also demonstrate that the credibility of women is questioned and that they perceive an uncaring attitude.

Halldòrsdottir (2008) argued that the value of visualizing or “seeing” the recipient of care in relation to her/his inner and outer context is very important. The interviewed women in this study gave no examples of such encounters with healthcare professionals. Discrediting patients leads to a risk that they will not receive optimal care, as well as flaws in documentation, assessment, and continuity of care. To cause the woman to suffer always implies violation of her dignity, failure to confirm her full worth as a human being, and denial of one's own holiness. The women narrated how they had exposed themselves without getting any protection. This experience caused unendurable suffering for the women, something that was also shown by Häggblom and Möller (2007).

In families where women are not economically disadvantaged, men may still resort to physical violence to reassure their masculine identity—violence which can be initiated by a man's need to compensate for the loss of esteem they experience when their economic ability is less than their spouses (Karakurt & Cumbie, 2012). Despite the fact that Sweden is one of the world leaders in terms of equal opportunities, it is surprising that the women in our study were seen as being in the way, a nuisance, and creating unnecessary problems. However, this can be interpreted as relative equality, but that the patriarchal structure still remains. Gender inequality in patriarchal social systems ensures that men have more resources available to them than women (Acker, 2006); likewise, society imparts men with a sense of entitlement to control women within their intimate relationships. And, since healthcare is a part of society, patriarchal structures also remain here. According to Häggblom and Möller (2007), IPV is still a hidden public health issue and may be underestimated by family members, politicians, health workers, social service, the police, the media, and other institutions. The issue remains a women's rights issue, also in Sweden.

Early intervention by healthcare staff can alleviate suffering, reduce the risk of post-traumatic stress, and create better conditions for the female victims of IPV to return to a normal life. Several studies have demonstrated that IPV has negative long-term health consequences for the victims (Campbell & Soeken, 1999; Campbell et al., 2002; Haddad et al., 2011). Instrumental behaviors on the part of healthcare professionals made the women in the present study feel like objects. According to earlier studies, IVP is also a financial burden on society due to the cost associated to both acute and long-term effects for the victims (Envall et al., 2006). There is a potential risk that women who experience IPV are unlikely to seek help due to lack of trust in the healthcare system. There is also a lack of knowledge among many nurses and other healthcare professionals about women's lived experience of IPV and the care provided following the violence (Stenson, 2004). The present study highlights the necessity for healthcare professionals, social workers, and the police to improve their knowledge about IVP and be aware of the risk of women of being retraumatized in their encounters with healthcare professionals and authorities. This is important in order to play an active role in the assessment of the quality of the help and care provided.

### Methodological considerations

The phenomenological hermeneutic method in the present study facilitated identification of the women's experiences of abuse and their encounter when seeking help. The themes that emerged from the narratives in the present study were reflected upon and found to validate the naive understanding. Although the study sample was small, the findings and knowledge derived can be useful when caring for women who have experienced IPV. The women who participated in this study exhibited great variation and their description of experiences was rich and nuanced. It is plausible that other women in the same situation will have similar experiences and that the results can therefore be transferred to different settings.

All participants were recruited from a shelter in a big town and therefore also their experiences from emergency departments came from big hospitals. It might have been other experiences reported from women in small towns and hospitals, so the transferability of the findings concerning such emergency departments has to be made with caution. There is also a risk that the authors’ preunderstanding and presumption have affected the interpretation of the transcribed text in a negative way. However, all three authors have separately returned to the women's statements in all of the phases of analysis, and the quotations make it possible for the reader to interpret what is mediated.

## Conclusions

The present findings raise awareness of how healthcare professionals, social workers, and the police perceive women who are victims of IPV. Satisfaction with the meeting of different professions can be regarded as a quality indicator of the care and support given to these women. To change negative attitudes and behaviors toward female victims of IPV requires that all involved professions develop their knowledge of causes and impacts of domestic violence. Women experiencing IPV need a realistic assessment of their legal situation. By acknowledging female IPV victims, it is possible to give them a voice and empower them to recover. Information about the various forms of care and assistance available can give the women a sense of control over the situation and enable them to decide what to do.

## References

[CIT0001] Acker J (2006). Class questions: Feminist answers.

[CIT0002] Battaglia T, Finley E, Liebschultz J (2003). Survivors of intimate partner violence speak out—trust in patient–provider relationship. Journal of General Internal Medicine.

[CIT0003] Campbell D, Sharps P, Gary F, Campbell J. C, Lopez M (2002). Intimate partner violence in African American women. Journal of Issues in Nursing.

[CIT0004] Campbell J. C, Soeken K (1999). Forced sex and intimate partner violence: Effects of women's risk women's health. Violence Against women.

[CIT0005] Choi S. Y. P, Ting K (2008). Wife beating in South Africa: An imbalance theory of resources and power. Journal of Interpersonal Violence.

[CIT0006] Dahlberg L. L, Krug E. G, Krug E. G, Dahlberg L, Mercy J. A, Zwi J, Lozano R (2002). Violence: A global public health problem. World report on violence and health.

[CIT0007] Envall E, Eriksson A, Marnell A (2006). The costs of violence against women.

[CIT0008] Eriksson K (2006). The suffering human being.

[CIT0009] Glass N, Wellings K, Branigan P, Mitchell K (2000). Discomfort, discord and discontinuity as data: Using focus groups to research sensitive topics. Culture, Health & Sexuality.

[CIT0010] Haddad L. G, Shotar A, Younger J. B, Alzyoud S, Bouhaidar C. M (2011). Screening for domestic. Violence in Jordan: Validation of an Arabic version of a domestic violence against women questionnaire. International Journal of Women's Health.

[CIT0011] Häggblom A. M, Möller A. R (2007). Interviews with battered women. International Journal of Qualitative Studies on Health and Well-being.

[CIT0012] Halldòrsdottir S (2008). The dynamics of the nurse–patient relationship: Introduction of synthesized theory from the patients’ perspective. Scandinavian Journal of Caring Sciences.

[CIT0013] Karakurt G, Cumbie T (2012). The relationship between egalitarianism, dominance and violence in intimate relationships. Journal of Family Violence.

[CIT0014] Kernic M. A, Wolf M. E, Holt V. L (2000). Rates and relative risk of hospital admission among women in violent intimate partner relationships. American Journal of Public Health.

[CIT0015] Kruttschnitt C, Ruback R. B, Weiner N. A (1995). Violence by and against women: A comparative and cross-national analysis. Interpersonal violent behaviors. Social and cultural aspects.

[CIT0016] Lindseth A, Norberg A (2004). A phenomenological hermeneutical method for researching lived experiences. Scandinavian Journal of Caring Sciences.

[CIT0017] National Center for Injury Prevention and Control (2003). Costs of intimate partner violence against women in the United States.

[CIT0018] Payne B. K (2007). Victim advocates’ perceptions of the role of health care workers in sexual assault cases. Criminal Justice Policy Review.

[CIT0019] Ricouer P (1976). Interpretation theory: Discourse and the surplus of meaning.

[CIT0020] Ricouer P (2005). Hermeneutic and the human sciences.

[CIT0021] Stark E (2010). Do violent acts equal abuse? Resolving the gender parity/asymmetry dilemma. Sex Roles.

[CIT0022] Stenson K (2004). Men's violence against women—a challenge in antennal care (Doctoral dissertation).

[CIT0023] The Swedish Institute (2011). Gender equality: The Swedish approach to fairness. http://www.sweden.se/eng/Home/Society/Equality/Facts/Gender-equality-in-Sweden.

[CIT0024] The Swedish National Council for Crime Prevention (2009). Victimization, fear of crime and public confidence in the criminal justice system: Report no 2010:2. http://www.bra.se/download/18.cba82f7130f475a2f180002016/1309946307101/2009_12.

[CIT0025] Watson S. D (2010). Relationship of vulnerability to coercive control and intimate partner violence (IPV) among Latinas confluence of risk factors for IPV (Dissertation, University of Miami).

[CIT0026] World Medical Association (2013). World Medical Association Declaration of Helsinki. http://www.isdbweb.org/pages/34.

